# Complementary substrate-selectivity of metabolic adaptive convergence in the lignocellulolytic performance by *Dichomitus squalens*

**DOI:** 10.1111/1751-7915.12134

**Published:** 2014-06-03

**Authors:** Jin Seop Bak

**Affiliations:** Department of Chemical and Biomolecular Engineering, Advanced Biomass R&D Center, KAISTDaejeon, 305-701, Republic of Korea

## Abstract

The lignocellulolytic platform of the wood-decaying organism *Dichomitus squalens* is important for production of biodegradable elements; however, the system has not yet been fully characterized. In this study, using statistical target optimization, we analysed substrate selectivity based on a variety of *D. squalens* metabolic pathways using combined omics tools. As compared with the alkali-lignin (AL) programme, the rice straw (RS) programme has the advantage of multilayered signalling to regulate cellulolytic-related genes or to connect their pathways. The spontaneous instability of the AL programme was accelerated by harsh starvation as compared with that of the RS programme. Therefore, the AL programme converged on cellular maintenance much easier and more rapidly. However, regardless of external substrate/concentration type, the compensatory pattern of the major targets (especially peroxidases and growth regulators) was similar, functioning to maintain cellular homeostasis. Interestingly, ligninolytic-mediated targets under non-kaleidoscopic conditions were induced by a substrate-input-control, and especially this mechanism had an important effect on the early stages of the biodegradation process. This optimized target analysis could be used to understand lignocellulolytic network and to improve downstream efficiency.

## Introduction

Biofuel production from lignocellulolytic biomass is actively being explored as an alternative energy source due to the increasing cost and inevitable depletion of conventional crude oil and due to global warming caused by the consumption of fossil fuels (Lynd *et al*., 1991; Kwok, 2009). However, the physicochemical recalcitrance of biomass fabric (especially lignin) is a critical obstacle for the enzymatic hydrolysis of biomass; therefore, relevant physicochemical pretreatments are essential before ethanol fermentation (Himmel *et al*., 2007; Sanderson, 2011). Recently, in order to address shortcoming of classical processes (Agbor *et al*., 2011), research on lignocellulolysis has refocused on in-depth biological methodologies with more environmentally friendly steps (Dashtban *et al*., 2009; Bak *et al*., 2010).

Few microorganisms have evolved to synthesize various oxidative enzymes with ligninolytic capabilities. In particular, the ligninolytic capabilities of the white-rot basidiomycete *Dichomitus squalens* have been extensively studied due to the spontaneous stability of oxidative enzymes (especially laccase and peroxidase) and the real possibility of percent theoretical yields (Cullen and Kersten, 2004; Bak *et al*., 2010). Recently, the 40–50 Mb *D. squalens* genome was identified (Floudas *et al*., 2012) and published by the Joint Genome Institute of the US Department of Energy (http://genome.jgi-psf.org/programs/fungi/fungal-projects.jsf), but the regulatory and metabolic programmes involved are not well understood. More importantly, the lignocellulolytic process of *D. squalens* involves a long-term fermentation process, representing an environmentally adapted system (Bak *et al*., 2010). Thus, metabolic profiling based on substrate specificity may be needed for the elucidation of simultaneous biodegradation networks. In whole-cell systems with *D. squalens*, no reports have yet attempted to verify data profiles (either upstream or downstream) based on a statistically optimized system of key targets. Furthermore, no studies have attempted to determine the complementary mechanisms of *D. squalens* ligninolysis (or cellulolysis) by simultaneously analysing expression data at the omics level.

Here, we applied combined polyomics (proteomics and metabolomics) on dystrophically cultivated *D. squalens* with two renewable substrates (rice straw and alkali-lignin) under optimized conditions. Our goal was to achieve improved substrate specificity in a *D. squalens* system that could help exploit its biodegradation abilities. An in-depth understanding of these processes can contribute to the improvement of biodegradability for bioethanol fermentation and is imperative for the development of downstream platforms.

## Results and discussion

### Classification of functional targets

**Figure 1:**
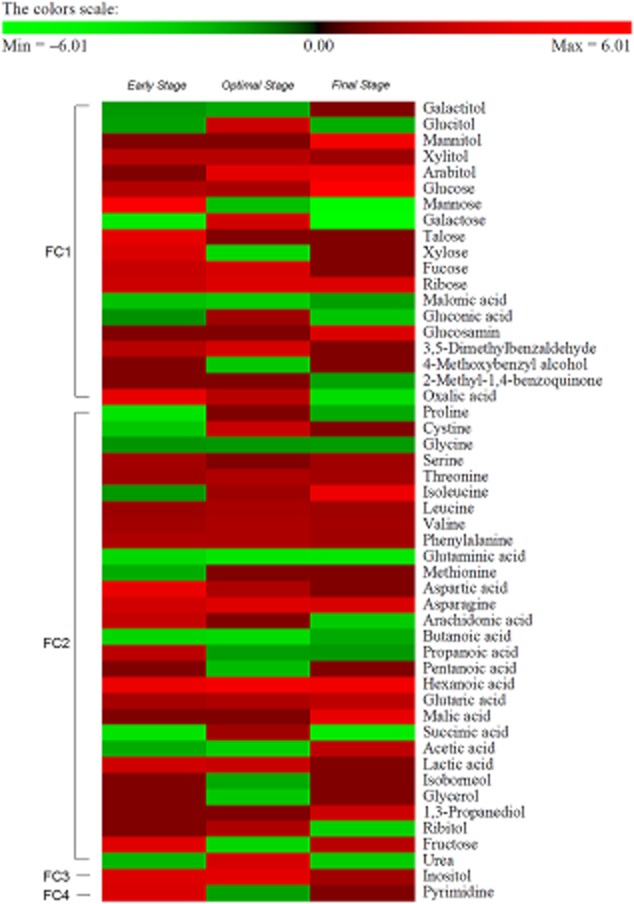
Downstream profiles in optimized *D. squalens* grown on lignocellulosic substrates. Hierarchical classification of 50 metabolites showing significant variation in regulation with 0.01 ≤ *P* < 0.05 and |fold| > 2 in AL group. Expression profiles from culture grown on AL for 7 (early), 15 (optimal) and 30 (final) days. Functional clustering of significant targets was determined as molecular biological functions released by the US Department’s Joint Genome Institute and National Institute of Standards and Technology. The scale reflects the logarithmic unit as compared with the negative control (without external substrates).

**Figure 2:**
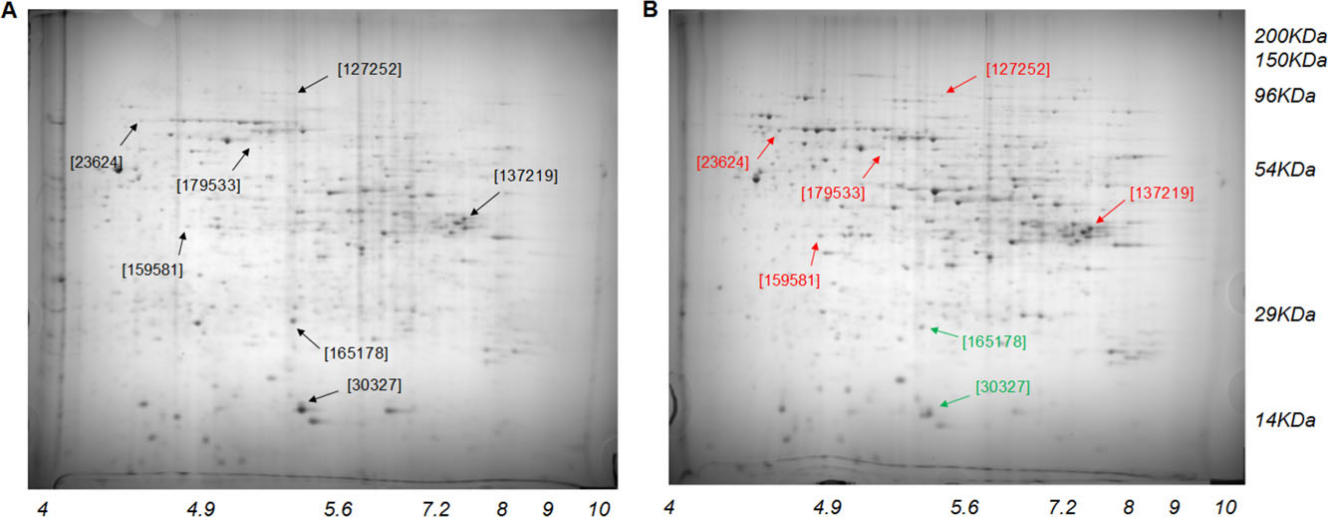
Open map of *D. squalens* proteome. The fungus was independently cultured for 15 days (optimal stage) on (B) alkali-lignin. The (A) negative control was grown without external substrates. Seven points on the 2-DE images show core proteins with *P* < 0.05 and |fold| > 2, i.e. meaning significantly lower (*green*) or higher (*red*) regulations compared with the responding proteins in control. The identity of adopted genes encoding proteins of *D. squalens* was named based on US Department’s Joint Genome Institute.

Based on the complementary collaboration of systemized polyomics profiles, we could infer the regulation of predominant controllers. First, after eight repeated experiments under the best conditions, we identified a total of 50 targets in alkali-lignin (AL) programme with |fold| > 2 and 0.01 ≤ *P* < 0.05 compared with the control (no substrate; either rice straw (RS) or AL), and they were grouped via hierarchical clustering into four distinct functional clusters [FC1; ligninolytic (or lignocellulolytic) mechanism (38.0%), FC2; metabolic transport and fundamental metabolism (58.0%), FC3; signal transduction and defence (2.0%), and FC4; cell growth and regulatory system (2.0%)] based on the public database (Fig. 1). Next, after six repeated observations, we obtained proteome profiles at optimal stage (15 days; percent maximum yields) of fungal fermentation to verify the downstream data. In two-dimensional gel electrophoresis (2-DE) reference map, seven encoded proteins showed predominant expression variations (more than |twofold|; *P* < 0.05) compared with the control (Fig. 2). Similar to downstream analysis, the selected proteins divide into five sections (FC1–4 and unknown), and detail information regarding the identification was checked by the open database (Supporting Information Table S1).

### Specific control in substrate-selective metabolism

**Figure 3:**
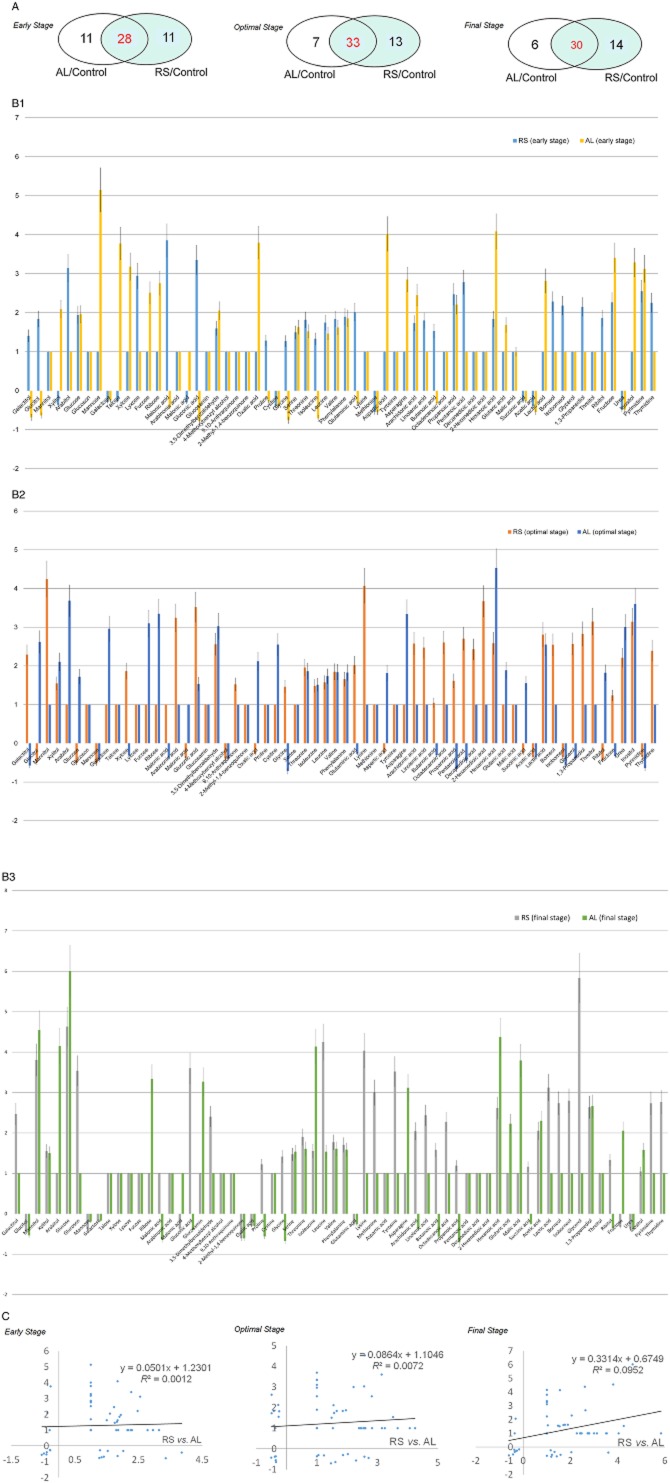
Comparative analysis of target metabolome in *D. squalens* cultured using alkali-lignin and rice straw as substrates. Metabolomic profiles from cultures grown on AL or RS for 7 (early), 15 (optimal) and 30 (final) days. (A) Overlap in the predominant metabolites between the *D. squalens* RS and AL groups. (B and C) Analysis of vertical bar plotting and linear regression (*R*^2^) between the RS and AL cultures based on the logarithmic intensities of significant 64 metabolites (50 metabolites in AL and 58 metabolites in RS) of *D. squalens*.

**Figure 4:**
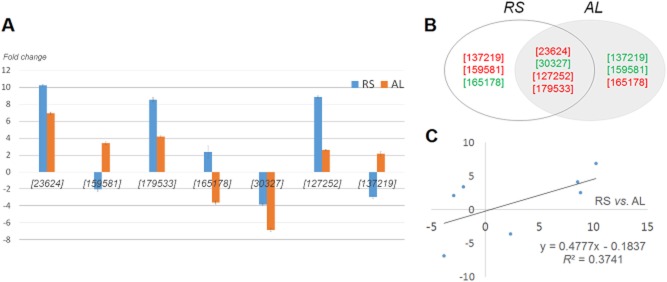
Contrastive analysis of significant proteome in *D. squalens* cultured using alkali-lignin and rice straw. Proteomic profiles from cultures grown on AL or RS for 15 days (optimal stage). In order to interpret the interrelationship between the functional targets, both 2D vertical bar-plot imaging (A) and linear regression (C) were carry out under different substrates (RS versus AL). (B) Overlap in the predominant proteins between the two groups (RS versus AL).

**Figure 5:**
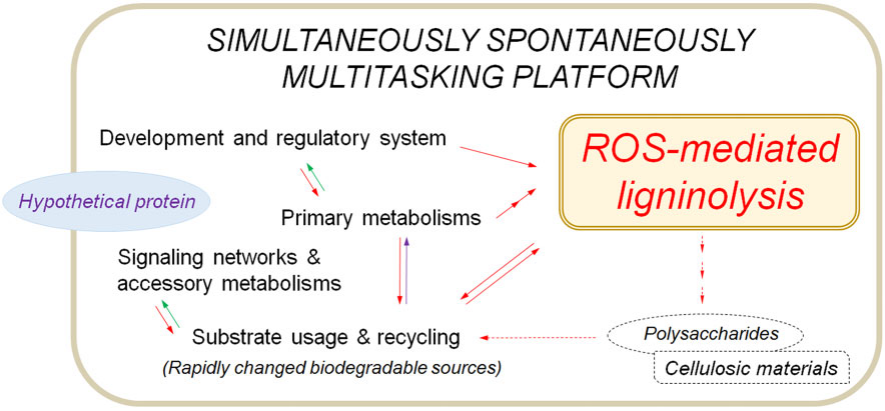
Schematic outline of simultaneously spontaneously multitasking platform in *D. squalens* system. The reactive oxygen species-mediated peroxidation programme is the keystone of *D. squalens* ligninolysis. The dotted line announces the substrate-specific regulation of *D. squalens* grown under optimized deconstruction with rice straw as an external substrate. The arrows on the map are designated as purple (upregulated and downregulated), red (upregulated) and green (downregulated) based solely on the polyomics profiles.

Cellulose exhibits an intricate structure composed of lignin and hemicellulose, in the form of lignocellulose. Thus, prior to the simultaneous saccharification and fermentation (SSF), the examination of AL-based profiling (only lignin-culture) are essential for the understanding of the expanded ligninolytic (or lignocellulolytic; RS-based profiling) cascades. We compared our AL dataset with RS dataset to understand the constitutive patterns of lignocellulolytic targets regulated in *D. squalens* biosystem, depending on the lignocellulosic composition. Regardless of external substrates, interestingly, although the apodictic profiles of the AL programme did not coincide with those of the RS programme, the overall correlation was reasonably similar, particularly in proteome profiles (Figs 3 and 4). Especially, the fundamental metabolisms of most targets based on ligninolytic cascades were very similar, probably due to their roles (for homeostasis and stability) in a complementary manner (Fig. 5). On the other hand, factors related to intracellular development programmes showed further compensating power in AL (Table 1). In detail, under optimal AL culture, we observed the limitation of essential intermediates (especially malonate) along with lower concentration of the cellulosic by-products (especially xylose, mannonic acids and gluconic acid), compared with those of RS culture (Fig. 3B and Table 2). On the other hand, after optimal stage of RS culture, we detected an elevated production of growth-mediated precursors (Asn, Asp, ribose and succinate) over AL culture. Additionally, the levels of the by-products (arachidonate, 2-hexenedioate, butanoate and pentanoate) related to polyhydroxyalkanoates (PHAs) metabolism were increased in RS. As we observed in metabolome data, a variation in the expression of oxidoreductive hydrolases was shown to be caused by different substrate-selectivity, and this relatively lower induction [alpha/beta hydrolase (ABH) fold-3, glycoside hydrolase, family 28 (GH28) and polysaccharide deacetylase (PDA)] in AL culture (Fig. 4A and Table 1). On the other hand, in some growth-related and unknown targets (Ras GTPase and hypothetical protein), we saw lower expression in RS compared with AL.

**Table 1:** List of key proteins that exhibit dominant change of expression during optimized biodegradation under the presence of different materials (RS versus AL)

JGI ID^a^	Functional classification^a^	Molecular function^a^	Change in expression^b^	|Fold| (*P* < 0.05)
				RS: AL^c^
179533	FC1 and FC2	Alpha/beta hydrolase fold-3	Upregulated in both	2.1
165178	FC1 and FC2	Glycoside hydrolase, family 28	Upregulated in RS/Downregulated in AL	8.3
127252	FC1 and FC2	Polysaccharide deacetylase	Upregulated in both	3.4
159581	FC3 and FC4	Ras GTPase	Downregulated in RS/Upregulated in AL	0.14
137219	Unclassified	Hypothetical protein	Downregulated in RS/Upregulated in AL	0.16

a The classification and putative function of the proteins were assigned based on the US Department’s Joint Genome Institute database.

b Relative expression of AL and RS compared with that of control (no substrate).

c Relative fold change ratio of the targets between AL and RS based on RS level.

**Table 2:** List of core metabolites that exhibit predominant change of expression during optimized *D. squalens* degradation under the presence of different lignocellulosic materials (RS versus AL)

Target	Functional classification^a^	Stage^b^	Change in expression^c^	|Fold| (*P* < 0.05)
				RS: AL^d^
Galactitol	FC1	Optimal	Upregulated in RS/Downregulated in AL	4.0
Glucitol	FC1	Optimal	Downregulated in RS/Upregulated in AL	0.19
Mannitol	FC1	Optimal	Upregulated in RS/Notexpressed in AL	4.2
Arabitol	FC1	Early	Upregulated in RS/Notexpressed in AL	3.2
Glucose	FC1	Optimal	Downregulated in RS/Upregulated in AL	0.29
Glucoson	FC1	Final	Upregulated in RS/Notexpressed in AL	3.5
Xylose	FC1	Optimal	Upregulated in RS/Downregulated in AL	5.9
Fucose	FC1	Optimal	Notexpressed in RS/Upregulated in AL	0.32
Ribose	FC1	Optimal to Final	Notexpressed in RS/Upregulated in AL	0.30 to 0.30
Mannonic acids	FC1	Early	Upregulated in RS/Downregulated in AL	9.3
Arabinonic acid	FC1	Optimal	Upregulated in RS/Notexpressed in AL	3.2
Malonic acid	FC1	Early to Optimal	Downregulated in RS/Notexpressed in AL	0.30 to 0.37
Gluconic acid	FC1	Final	Upregulated in RS/Downregulated in AL	9.1
Glucosamin	FC1	Final	Notexpressed in RS/Upregulated in AL	0.31
Oxalic acid	FC1	Optimal	Downregulated in RS/Upregulated in AL (Downregulated at Final in both)	0.13
Proline	FC2	Early	Upregulated in RS/Downregulated in AL	4.7
Glutaminic acid	FC2	Early to Optimal	Upregulated in RS/Downregulated in AL	7.6
Lysine	FC2	Optimal to Final	Upregulated in RS/Notexpressed in AL	4.1 to 4.0
Methionine	FC2	Final	Upregulated in RS/Notexpressed in AL	3.0
Aspartic acid	FC2	Optimal	Downregulated in RS/Upregulated in AL	0.14
Tyrosine	FC2	Final	Upregulated in RS/Notexpressed in AL	3.5
Asparagine	FC2	Optimal to Final	Notexpressed in RS/Upregulated in AL	0.30 to 0.32
Arachidonic acid	FC2	Final	Upregulated in RS/Downregulated in AL	5.5
Butanoic acid	FC2	Early to Optimal	Upregulated in RS/Downregulated in AL	4.8 to 3.4
Pentanoic acid	FC2	Optimal	Upregulated in RS/Downregulated in AL	6.0
2-Hexenedioic acid	FC2	Optimal	Upregulated in RS/Notexpressed in AL	3.7
Succinic acid	FC2	Optimal	Downregulated in RS/Upregulated in AL	0.17
Lactic acid	FC2	Final	Upregulated in RS/Notexpressed in AL	3.1
Glycerol	FC2	Optimal	Upregulated in RS/Downregulated in AL	6.7
Threitol	FC2	Optimal	Upregulated in RS/Notexpressed in AL	3.2
Ribitol	FC2	Optimal	Downregulated in RS/Upregulated in AL	0.24
Fructose	FC2	Optimal	Upregulated in RS/Downregulated in AL	3.8
Inositol	FC3	Early	Notexpressed in RS/Upregulated in AL	0.30

a The classification of the targets were assigned based on the released databases from National Institute of Standards and Technology and US Department’s Joint Genome Institute.

b Culture period prior to analysis when the significant change was observed.

c Relative expression of AL and RS compared with that of control (no substrate).

d Relative fold change ratio of the targets between AL and RS based on RS level.

### Non-specific system independent of external substrates

**Figure 6:**
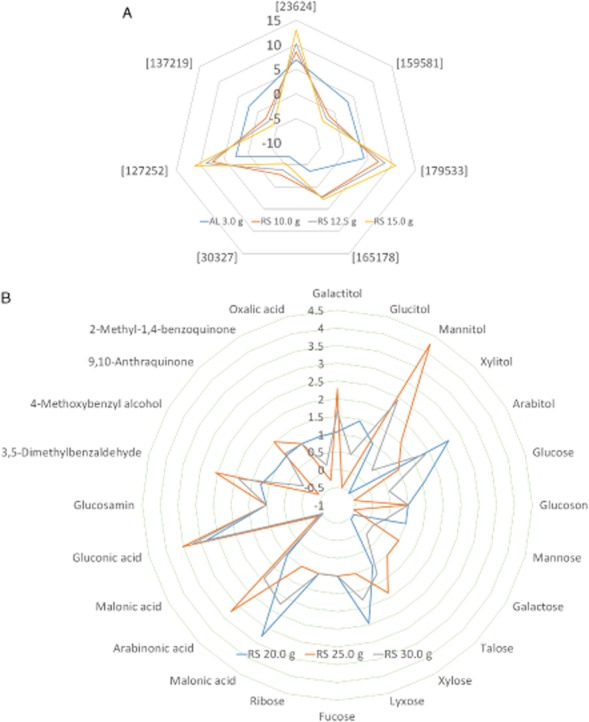
Comparative 2D radial-plot imaging of core targets involved in lignocellulolytic mechanism (FC1) based on the culture type of *D. squalens* grown on substrates for 15 days. These plots indicates a combined display of either substrate type (AL and RS) or concentration type (AL 1.1 g, RS 4.0 g, RS 5.0 g, and RS 6.0 g). These (A) 7 proteins (|fold| > 2 and *P* < 0.05) and (B) 24 metabolites (0.01 ≤ *P* < 0.05 and |fold| > 2) were selected with significant variation (based on the logarithmic intensities) as compared with the control (no substrate). Putative functions of the target proteins are shown in Table 2.

Through our closed *D. squalens* system without supplying additive nutrients (Figs 1 and 2), regardless of the absence of lignin-related substrates (i.e. negative control), the peroxidative radical-mediated depolymerization (by hyperaggressive degraders) of lignin complexes was developed from external factors (especially transiently supplied soluble cellulosic sugars or a rapidly reduced nitrogen source). These induction events were also reported to be similarly regulated in other species, but their activities under nitrogen-sufficient conditions were different from our patterns of lignocellulolytic capacity (Bak *et al*., 2009; Znameroski *et al*., 2012). More remarkably, independent of culture type (especially RS cultures), the overall correlation of FC1-targets was considerably analogous, especially in upstream (Fig. 6). It shows that cells would not consistently make any more aggressive ligninolysis, probably due to the homeostatic equilibrium for survival and growth. Furthermore, although the activation of conserved defence system is generally minor in all optimized *D. squalens* cascades, the system may major function as a decision-making programme to maintain the cellular stability in overall metabolic fluxes. Similarly, under harsh condition, the active transduction programme containing the biocommunication networks was significantly found in the cellular maintenance mechanism of the other white-rot fungal system (Matsuzaki *et al*., 2008; Yildirim *et al*., 2011).

### Extracellular evaluation and scale-up of *D. squalens* biosystem

Independent of concentration and type of substrates, key oxidative hydrolases would be reflected in increased powers, and their extracellular mechanisms led to the loss of core components (Table 3). In medium RS culture (here 12.5 g), we observed the best evaluations for industrial scale-up, compared with those of other cultures. Remarkably, we confirmed that the remained materials (especially recalcitrant lignins) in substrate are absolutely critical for the percent maximum yields. In cell wall-loosening platform by *D. squalens*, the peroxidative mechanisms made various cellulose-related enzymes more accessible to disrupted (or modified) substrates, and thus it could ever more increase the percent glucose yield (Chen and Dixon, 2007). For reference, our fungal-based system was lower than those of feedstock (> 80% of hydrolysis maximum) pretreated using conventional tools (Agbor *et al*., 2011). However, our system was superior to previous bio-based systems, in terms of both the percent productivity (< 50% of maximal glucose and ethanol) and the percent efficiency (time and cost) (Dashtban *et al*., 2009).

**Table 3:** Bottom-up results based on the culture-type in *D. squalens* biodegradation after optimal stage

Type	Amount (g/L, dry wt. basis)	Change of substrate components (g/L) (before/after)			Monomeric sugar (g/L)	Activity of extracellular enzymes (U/L)				Index of evaluation	
		lignin	glucan	xylan	Glucose^a^	Laccase MnP^b^	GLO^c^ AAO^d^	β-glucosidase CDH^e^	Xylanase	glucose (%)^f^	ethanol (%)^g^
AL	5.5 g	5.50/3.30	–	–	–	∼200	∼350	–	–	–	–
						∼1,200	< 40				
RS	30.0 g	5.91/3.56	10.70/7.48	3.24/2.16	< 0.24	∼250	∼380	∼140	∼200,000	∼60	∼60
						∼1,100	< 20	∼40			
RS	25.0 g	4.93/2.96	8.92/6.20	2.70/1.66	< 0.22	∼300	∼450	∼120	∼260,000	∼63	∼62
						∼1,500	< 40	∼40			
RS^h^	20.0 g	3.94/2.40	7.14/5.01	2.16/1.42	< 0.20	∼200	∼400	∼110	∼210,000	∼58	∼60
						∼1,300	< 30	∼30			

a soluble glucose from the biodegradable substrates during the SSF.

b manganese peroxidase.

c glyoxal oxidase.

d aryl alcohol oxidase.

e cellobiose dehydrogenase.

f theoretical maximum yield of glucose from the enzymatic hydrolysis.

g theoretical maximum yield of ethanol from the SSF.

h Bak *et al*., 2010.

### Step I: reactive oxygen species (ROS)-mediated degradation for open platform

ROS-mediated peroxidation (especially by hydrogen peroxide) are at the core of the ligninolytic mechanism by wood decaying fungi, and the oxidative metabolisms are continuously activated during the fungal degradation (Hammel *et al*., 2002). Regardless of culture type (AL or RS), the positive activation [especially via manganese peroxidase (MnP) and ABH] of ligninolysis broadly accepted in this *D. squalens* system (Fig. 4A and Table 1). In peroxidative mechanism, first, Mn(III) (induced by MnP) was reduced to Mn(II) by oxidized materials, and then had implemented a rotation system for the oxidation of lignin. Recently, the extracellular short modules (or versatile) of MnP cascades have focused on fungal ligninolysis (Salame *et al*., 2014). However, these cascades were not highly activated in our study. Additionally, under conditions of nitrogen restriction, the post-transcriptional regulation by manganese is directly involved in the induction and accumulation of MnP (Kamei *et al*., 2008). At the same time, under the activation of ligninolysis (Bugg, 2004), the alpha/beta hydrolase fold families had the supporting cascades in providing cells with the ability to deconstruct recalcitrant polymers. Furthermore, the redox cascade of biomineral complexes (especially ferric-oxalate and manganese-malonate) could contribute to promote the radical-based lignin-wall deconstruction (Table 2). Moreover, the participation of other inducible factors (e.g. hydroquinones) was positively activated in open cascades. Interestingly, more aggressive ligninolysis occurs via the oxidation of substrates by several oxidases (especially glyoxal oxidase, aryl alcohol oxidase and laccase), but no targets were found to exhibit significant changes in expression (|fold| < 2) in both cultures. However, we confirmed the extracellular activities of these enzymes (Table 3). As indirect proof of their extracellular activities, loss of lignin content was also induced by biodegradation. Consequently, the structural open of lignin by the radical-mediated cascades was shown to consistently reduce the crystallinity of substrates. For reference, the trend between percent sugar maximum and crystallinity index was confirmed to be negatively controlled (Bak *et al*., 2010), which suggests that the amorphous fraction of lignocellulose is sufficiently depolymerized by extracellular peroxidases.

### Step II: lignocellulolysis (or cellulolysis) for efficient bioenergy system

Despite the non-specific regulation of *D. squalens* programme, the presence of useful biodegradable components (especially cellulosic macromolecules; RS-culture system) had a noticeable effect on the downstream bioprocesses as well as the genetic expressions.

Unlike the general trends of ligninolytic enzymes, all carbohydrate-active enzymes (CAZys; here PDA and GH28) and their chaperons were found to show non-comparable activities (more powerful in RS than AL) because of substrate-specific activation on substrates with different compositions (Fig. 4A and Table 1). We believe that active-site attachment programme (especially via carbohydrate-binding modules/GHs) in enzyme–substrate interactions gives weight to the depolymerization of polysaccharides (especially pectin compounds). Especially, GH28’s function in cellulolytic programme are evolutionarily extended as multiple functions, such as rhamnogalacturonases and xylogalacturonan hydrolase in order to support their catalytic diversity and efficiency (Markovic and Janecek, 2001). Similar to our profiles, the induction of GH28s in both *Phanerochaete chrysosporium* (3 GH28; Vanden Wymelenberg *et al*., 2009) and *Aspergillus niger* (12 GH28; Martens-Uzunova *et al*., 2006) was reported to enhance the catalytic effects of other enzymes that hydrolyse polysaccharides in the presence of lignocellulosic materials. Remarkably, based on long-term biodegradation, adaptation on lignocellulose (relative to fermentable sugars) was better for *D. squalens* than for the ligninolytic fungus *Ceriporiopsis subvermispora* (Fernandez-Fueyo *et al*., 2012). The key difference with respect to the expression of CAZys appeared to be in the number of polysaccharide hydrolases (especially GH28), which is lower in *D. squalens* than in other species. No significant differences in other polysaccharide-related families were detected, suggesting that the additional GH28 enzymes in *D. squalens* have a significant influence on pectin hydrolysis. Due to our advanced biological pretreatment (via process optimization), we believe that optimal (or minimal) factors contributed to the more aggressive cellulolysis of external substrates. On the other hand, interestingly, core extracellular cytochrome CAZys (e.g. cellobiose dehydrogenase, beta-glucosidase and hemicellulases) did not exhibit major variations in expression (|fold| < 2) in all cultures (Fig. 4A). However, their extracellular activities were carefully predicted in the *D. squalens* system (Table 3). We could also predict the intervention and connectivity of the cellulosome in order to enhance the yield of biological pretreatment. Under the presence of cellulosic biomass, cellulosome complexes with high GH abundance are particularly powerful for the production of fermentable monomers (Shoham *et al*., 1999); monomeric sugars (i.e. glucose, < 0.2 g/L) rather than dimers or oligomers (e.g. cellobioses) were observed during the SSF, regardless of whether undegraded (control) or degraded RS was used (Table 3). In particular, an abundant supply of fatty acids (especially short-chain form; Table 1) released from PHAs are usefully used as hydrophobic binding motifs (for cellulosomes) in mediating biological deconstruction (Bolobova *et al*., 1994). Furthermore, in order to save the cost of fermentable substrates (especially monomeric sugars), either degradation (Escapa *et al*., 2012) or accumulation (Anderson and Dawes, 1990) of the PHAs were inextricably involved in fundamental and secondary metabolisms. For reference, regarding the inconsistent regulation of CAZys, regardless of target optimization, this possibility was previously observed in the functional genomic pools (Floudas *et al*., 2012), and the similar pattern was also identified in other wood-rot fungi (Martinez *et al*., 2004; Fernandez-Fueyo *et al*., 2012) and the other species (Stricker *et al*., 2008; Martinez *et al*., 2009; Tolonen *et al*., 2011).

After initial stage of fermentation, the expression of CAZys soon begin to predominate over the utilization (or reduction) of lignocellulosic (or cellulosic) hydrolysates (especially in RS culture after optimal stage) (Figs 3B and 5A), and also this cascade can seek somewhat similar to that of wood-degrading *Neurospora crassa* (Znameroski *et al*., 2012) and *P. chrysosporium* (Bak *et al*., 2009). Regarding carbon-based cascades, our profiles suggested that both pentose phosphate pathway and TCA-cycle are responsible for managing the controllers (intermediates and coenzymes) as required metabolic energy (or precursors) to a greater extent than the other fluxes (especially glycolysis). However, for example, in the mannitol pathway within glycolytic programme, they get involved in the regeneration of coenzyme factors [e.g. NADP(H)], which is important for the interactive interchange of metabolic cascades.

### Cellular development and signalling for metabolic adaptive convergence

Regardless of substrate type, *D. squalens* biodegradation sequentially deal with central synthetic routes of cofactors, such as electron accepters and flux controllers (especially binding-module Src homology-3) and growth precursors (e.g. ribose-5 phosphate and inositol), on intracellular multiple cascades (Figs 3B and 4A). In a view of anabolic proliferation, the growth-promoting signals can be accelerated the metabolic system via the de novo pyrimidine synthetic process, and thereby the rate-limiting performance of biodegradation help to coordinate production of nucleic acids for cell development and maintenance (Ben-Sahra *et al*., 2013), particularly early and final stages (Tables 1 and 2). This hints that short-term perturbations in the regeneration system of various modulators are stabilized via the complementary metabolic pathways. On the other hand, signalling controllers (especially Ras GTPase) related to intracellular regulatory and development showed further compensating capability in AL, and as a result, cells prevent the programmed cell death more strongly (Fig. 4A and Table 1). Especially, the control mechanism of relevant post-transcription as well as some signallers (e.g. Ras-superfamilies and G-proteins) is somewhat similar to that of the other species (Phillips *et al*., 2006; Yildirim *et al*., 2011). This result is that deficiency of energy source in form of polysaccharides demands the cells to require further stabilized system.

In downstream, it appeared that the metabolic products (especially succinate, Asn and Asp) released from deconstruction activities are not being properly utilized during the especially optimal stage (Fig. 3B and Table 2), since the majority of energy is devoted to ligninolytic performance instead of cell development and overgrowth. Additionally, the amphiphilic function of short-chains carboxylic acids (from PHAs) appears to be beneficial for the metabolic diversity as well as the fermentation efficiency (Bak *et al*., 2009).

Lastly, based on the distinct regulation of unknown targets (especially hypothetical proteins; Table 1), we believe that their active participation in the depolymerization of cellulolytic materials is barely suitable for enhancing the theoretical sugar yield. However, these unknown targets may affect the activation of core controllers within signalling events and may be important for growth-inducible metabolism.

## Conclusions

This research was the first to evaluate the specific utilization of plant biomass by *D. squalens* based on a polyomics approach. Importantly, through our study with both statistical optimization and continuous process analysis, large amounts of data were further reshuffled to the lignocellulolytic system, and the stable productivity of the closed *D. squalens* system was improved. Regardless of external substrates, the major role of CAZy systems via peroxidative mechanisms during biodegradation was broadly demonstrated. Interestingly, independent of metabolic origins, all networks appeared to operate in an open system based on non-specific regulation involving essential and secondary metabolism, signalling and development. This means that metabolic fluctuations in the regulation pattern have no significant effect on conserved system for efficient deconstruction of renewable biomass. More remarkably, regarding the scale-up evaluation, we confirmed that the major challenge is a matter of remained lignins rather than redundant celluloses. Lastly, this *D. squalens* biosystem may suggest a useful discipline within the field of downstream bioprocesses.

## Experimental procedures

### Fungal fermentation of recalcitrant substrates

Because the growth phase in different culture causes different circumstance (e.g. growth level, protein concentration and pH) of cell community, we carried out the whole observations after the optimization of ligninolytic peroxidases via statistical tools. Further details are provided in supporting information (Supporting Information Appendix S1). After the preprocessing steps (Supporting Information Appendix S1) for raw substrates, processed substrates (especially RS) were used as the model compound of lignocellulose. After addition of either 1.1 g AL or 5.0 g RS at 29°C and 150 r.p.m. for 30 days, *D. squalens* (CBS 432.34) was cultured in 200 ml of the optimized growth medium. Especially, pure AL (Sigma-Aldrich, St Louis, MO, USA) derived from plant biomass was used as the model compound of lignin. No substrate was added to the control samples. In order to understand the concentration-dependant profiles, two samples (6.0 g RS and 4.0 g RS) were simultaneously cultured under the same condition.

### Analysis of downstream chemicals and products

A 5975/7890 gas chromatography–mass spectrometry (Agilent Technologies, Waldbronn, Germany) that equipped a DB-5MS (J&W Scientific, Folsom, CA) was used for the identification and quantification of intracellular and extracellular products. Further details are provided in supporting information (Supporting Information Appendix S1). After eight biological replicates, the significance of predominant changes for the targets in each culture was verified using the paired *t*-test. The gaps among groups were verified using the unpaired *t*-test and analysis of variance. The statistical checks for downstream data were proceeded using SAS ver. 9.2 (SAS Institute, Cary, NC, USA) and SigmaStat 3.5 (Systat Software, San Jose, CA, USA). After the normalization of significant targets, hierarchical clustering analysis was applied to rearrange targets into functional clusters (Eisen *et al*., 1998). PermutMatrix ver. 1.9.3 (http://www.lirmm.fr/~caraux/PermutMatrix/) was the graphical analysis tool used to determine biases in the patterns of regulation across groups (Caraux and Pinloche, 2005).

### Complementary proteomic approach

After protein extraction from *D. squalens* pellets (Supporting Information Appendix S1), reference mapping of proteome was displayed using 2-DE to confirm the quantitative patterns of changing targets under AL or RS, as compared with the control. After six biological replicates of the cultures, the changed spots were identified by MS/MS sequencing and peptide mass fingerprinting based on the public database. Further details are provided in supporting information (Supporting Information Appendix S1). The statistical approaches of all gene-encoded proteins was systematically implemented using both SigmaStat and SAS.

### Index of evaluations of fungal biosystem

The extracellular activities of well-known targets involved in lignocellulose biodegradation were analysed during the fermentation based on previously reported methods (Supporting Information Appendix S1). According to the public protocols (http://www.nrel.gov/biomass/analytical_procedures.html), the change of three components (cellulose, hemicellulose and lignin) of RS were checked based on a dry weight basis (per 100 g substrate). Furthermore, the theoretical indexes of downstream evaluation were analysed based on the public protocols. All data shown are the mean values of triplicate experiments. Further details are provided in supporting information (Supporting Information Appendix S1).

### Confirmation of metabolic system from omics profiles

We applied a systematic approach to integrate data from omics pools. Detection of a series of downregulated targets hints towards a bottleneck or that the purported route is less favourable than alternative paths. Contrastively, their upregulation positively suggests a mainstream position. The confirmation in the regulation pattern was identified based on the literatures, by checking intermediates and via metabolic perturbations.

## Supporting Information

**Table S1.** Information of the adopted cellular proteins correlated with lignocellulolytic regulatory network during optimized *D. squalens* biodegradation. **Appendix S1.** Complementary substrate-selectivity of metabolic adaptive convergence in the lignocellulolytic performance by *Dichomitus squalens*.
