# Supplementary material for: Complementary substrate-selectivity of metabolic adaptive convergence in the lignocellulolytic performance by Dichomitus squalens
Source: Microb Biotechnol. 2014 Jun 3;7(5):434–45. doi: 10.1111/1751-7915.12134 (PMC4229324; doi:10.1111/1751-7915.12134)
Supplement: Supplementary file 1 — Table S1. Information of the adopted cellular proteins correlated with lignocellulolytic regulatory network during optimized D. squalens biodegradation. Appendix S1. Complementary substrate-selectivity of metabolic adaptive convergence in the lignocellulolytic performance by Dichomitus squalens. [file mbt20007-0434-sd1.doc]

Appendix S1

Complementary substrate-selectivity of metabolic adaptive convergence in the lignocellulolytic performance by *Dichomitus squalens*

Running Head: Substrate-specific metabolism by *D. squalens*

Jin Seop Bak

*Department of Chemical and Biomolecular Engineering, Advanced Biomass R&D Center, KAIST, 291 Daehak-ro, Yuseong-gu, Daejeon, 305-701, Republic of Korea*

Address correspondence to Jin Seop Bak, jsbwvav7@kaist.ac.kr

**Supplementary Experimental Procedures**

Unless otherwise noted, all starting materials were purchased from commercial suppliers and were used without further purification.

*Preparation of samples for experiments*

RS was harvested from Korea University Farm (Deokso, Korea), and then was milled using a cutting mill (MF 10, IKA, Staufen, Germany). RS-substrate was autoclaved at 121°C for 10 min prior to use. The solid content of substrate was found to be approximately 97.0% (w/w). According to the NREL protocols (http://www.nrel.gov/biomass/analytical_procedures.html), the amount of RS components revealed as 35.7% glucan, 3.7% mannan, 2.5% galactan, 10.8% xylan, 3.3% arabinan, and 19.7% lignin on a dry weight basis.

Similar to previous methods (1), medium containing both glucose (1 %, w/w) and yeast extract (0.5 %, w/w) was statistically optimized using Placket-Burman design with other nutrients such as vitamin solution, KH_2_PO_4_, CaCl_2_, MgCl_2_, and FeSO_4_. After the selection of target variables (yeast extract, CaCl_2_ and KH_2_PO_4_), they were subsequently optimized as 5.467, 0.066 and 10.230 g/L, respectively, using the Box-Behnken design. Actual activity from the experiments was checked as 4,990.8 ± 128.5 U/L (*p* < 0.05) in good correlation with the predicted value (5,200.8 U/L).

*Assay of well-known enzymes and scale-up evaluation*

Similar to previous procedures (1, 2), the activity of extracellular MnP was obtained by detecting a Mn (III)-malonate unit at 270 nm. In case of laccase, the amount of 2,6-dimethoxyphenol was checked at 469 nm (2). The phenolic compound was oxidized in a mixture that contained 0.2 mM 2,6-dimethoxyphenol and crude enzyme in 20 mM succinate buffer at pH 3.0. Furthermore, the activity of GLO was confirmed by monitoring the formation of radicals (here H_2_O_2_) using a modified peroxidase-coupled assay (3–5). Additionally, AAO was analyzed by the oxidation of VA at 310 nm (3, 6, 7).

The activities of key CAZys (here β-glucosidase and CDH) were analyzed based on the previously confirmed methods (8). The value of β-glucosidase was measured by monitoring the separation of p-nitrophenyl from p-nitrophenyl-β-D-glucoside at 400 nm for 5 min. The extracellular CDH activity was also assayed by the reduction of cytochrome c (12.5 μM) at 550 nm in the presence of cellobiose (100 μM). Additionally, the regulation of cytochrome c in CDH was identified via 2,6-dichlorophenolindophenol activity (1: 1.54 unit). Regarding the prediction of hemicellulolytic power (here xylanase activity), 1 mL mixture containing 0.5 mL of diluted supernatant of broth and 0.5 mL of 1–2% birchwood xylan (Sigma-Aldrich, St. Louis, MO, USA) in 0.05 M sodium citrate buffer (pH 4.8) was analyzed for 30 min at 50^o^C, reducing sugars were checked by the DNS protocol (http://www.nrel.gov/biomass/analytical_procedures.html). After fungal biodegradation, the fermentability (eq. 1) and digestibility (eq. 2) were indicated as a percentage of the theoretical maximum of products obtained from substrates.

(eq. 1)

(eq. 2)

*Systematic preprocessing methodology for downstream analysis*

Independently of substrate-type (AL or RS), fungal-cell separation from the biodegrading solution was executed as following: 5 ml of sample was injected into 26 ml of cold solution containing 60% (v/v) methanol buffered with 70 mM HEPES at pH 7.5. This was then kept at –40°C in a dry ethanol/ice bath. The mixture was cooled for 3 min and centrifuged at 5,000×*g* for 5 min at 0°C. After the centrifugation, the mixture was kept at –20°C. The cell-mass was determined to be approximately 30 mg (dry wt), after the residues were dried by using a vacuum-drying oven at 55°C. Intracellular metabolome was extracted from the mycelia pellets in 5 ml of 75% (v/v) boiling absolute ethanol buffered with 0.25 M HEPES at pH 7.5, and the mixture-solution was maintained for 3 min at 80°C. After cooling the solution on ice for 3 min, it was dehydrated using Speed Vac Plus (SC110A, Savant Instruments, Holbrook, NY) at room temperature (if possible 25°C). In order to analyze extracellular metabolome, after filtration through a 0.2-μm PVDF filter, the supernatant from the culture broths was evaporated to dryness under vacuum with the Speed Vac Plus at room temperature (below 25°C). After drying, all metabolome samples were resuspended in 80 μl of methoxyamine hydrochloride solution in pyridine (2 g/100 ml) and kept for 90 min at 30°C. Eighty milliliter of *N*-Methyl-*N*-(trimethylsilyl)trifluoroacetamide was then added to each sample, followed by incubation for 30 min at 37°C. Importantly, monomeric L-sorbose (62.5 nmol per sample) was added as the internal standard.

*Qualification and quantification of downstream chemicals and products*

The total ion chromatograms (TICs) using the GC-MS was performed to understand the expression patterns of *D. squalens* metabolome on RS-group as compared to those of AL-group. Here helium gas as the mobile-phase was used at 1 ml He/min. In detail, the injection volume and split ratio were 1 μl and 1:100, respectively. The GC-MS was operated in scan mode in the range of 50–550 amu. The gradient step used for the GC-MS analysis was as follows: 70°C for 5 min, 10°C/min to 179°C, 0.5°C/min to 180 °C with holding for 2 min, 10°C/min to 220°C with holding for 1 min, 2.5°C/min to 265°C with holding for 1 min, 10°C/min to 280°C with holding for 1 min, 1°C/min to 290°C, and 10°C/min to 300°C. A respectable number of peaks were sequentially obtained, and of the roughly 200 metabolites checked by spectral-matching against the public database of both the NIST research library (http://www.nist.gov/srd/nist1a.htm) and the DOE-JGI database (http://genome.jgi.doe.gov/); approximately 100 chemical compounds have been identified in at least 75% of subjects in all data profiles. To reduce possible analytical errors, the retention-times were controlled using internal standards (9). Furthermore, the integration of each peak obtained from the TIC normalized using authentic reference compounds were applied for the quantitative comparison of each metabolite.

*Fundamental procedure for analysis of fungal proteome*

In detail, regardless of external substrate (AL or RS), fungal pellets obtained from 15-day-old cultures were washed twice in ice-cold PBS and sonicated for approximately 10 sec with a Bandelin Sonoplus HD 200 (Bandelin Electronic, Berlin, Germany). The pellets were then maintained overnight at 4°C after the addition of 10 ml of 10% (w/v) trichloroacetic acid per cell-mass (mg), and they were then centrifuged at 15,000×*g*. Acetone was added to the resultant pellets, and the mixture-solution was kept for 1 h at 4°C. After centrifugation at 15,000×*g* for 1 h at 15°C, the dried pellets were dissolved in a lysis solution composed of 7 M urea and 2 M thiourea containing 4% (w/v) CHAPS, 1% (w/v) DTT, 2% (v/v) pharmalyte, and 1 mM benzamidine. Protein extraction was carried out by vortexing for 1 h at room temperature. After centrifugation at 15,000×*g* for 1 h at 15°C, the soluble fraction was made for 2-DE.

In order to normalize the loading amount in each sample, the total concentration of protein was determined by a Bradford method (10). IPG dry strips (pH 4–10 NL, 24 cm; Amersham Biosciences, NJ) were equilibrated for 12–16 h with 7 M urea and 2 M thiourea containing 2% (w/v) CHAPS, 1% (w/v) DTT and 1% (w/v) pharmalyte and then loaded with 200 µg of samples. IEF was carried out at 20°C using a Multiphor II electrophoresis unit (Amersham Biosciences). For the first dimension separation, the voltage was vertically increased from 150 to 3500 V over 3 h for the sample entry, and kept at 3500 V for a total of 96000 Vh. Before the second dimension, the strips were incubated in an equilibration buffer (pH 6.8; 50 mM Tris-HCl, 6 M urea, 2% (w/v) SDS, and 30% (w/v) glycerol) for 10 min, and then washed with 1% (w/v) DTT. After the incubation, the strips were also washed with 2.5% (w/v) iodoacetamide. Equilibrated strips were inserted onto SDS-PAGE gels (20 × 24 cm, 10–16%), and the SDS-PAGE was performed using a Hoefer DALT 2D system (Amersham Biosciences). The 2-DE gels were worked at 20°C for 1700 Vh, and then stained with CBB G250. Quantitative analysis of computerized images was carried out using PDQuest software ver. 7.0 (BioRad, Hercules, CA). Key spots with significant expression variation (|fold| > 2) under RS as compared to the expression level of AL proteome were selected for analysis.

*Proteomic data analysis*

Focused spots in 2-DE gels were enzymatically digested using modified porcine trypsin method (11). Segregated gels were washed with 50% (w/v) aqueous ACN to remove remnants (SDSs, salts, and stains). They were subsequently dried to remove solvents and were rehydrated with trypsin (8–10 ng/μl), then incubated for 8–10 h at 37°C. The degradation of cellular proteins were immediately stopped by adding 5 μl of 0.5% TFA. Tryptic peptide chains were recovered by mixing the byproduct mixture from the extractions of gel pieces with 50% (w/v) ACN. The mixture was desalted and concentrated using C_18_ZipTips (Millipore, Bedford, MA), and then eluted in 1–5 μl ACN. An aliquot of this solution was mixed with the same volume of a saturated solution of CHCA in 50% (w/v) ACN, and 1 μl of the mixture was spotted onto a test plate.

Proteomic analysis via chemically assisted fragmentation was performed using an Ettan MALDI-TOF (Amersham Biosciences). Peptide chains were evaporated with N_2_ laser at 337 nm by using a delayed extraction methodology. They were accelerated with a 20-kV injection pulse for the check of flight time. Each spectrum showed the accumulated average values of 300 laser shots. ProFound (http://129.85.19.192/profound_bin/WebProFound.exe) was used as an expert system for searching a protein sequence collections with PMF. Significant spectra were calibrated with trypsin auto-digestion ion-peaks at m/z 842.510 and 2211.104 used as the internal reference compounds. In order to improve the accuracy of identification, the selected spots were simultaneously analyzed using either a Voyager-DE STR MALDI-TOF (Applied Biosystems) or a 4700 Proteomics Analyzer MALDI-TOF/TOF (Applied Biosystems). When necessary, the samples were once more desalted using the C_18_ZipTips, and then they were eluted directly with 5 mg/mL of CHCA in 60% (w/v) ACN/0.1% (w/v) TFA onto a MALDI plate. All MS spectra were recorded in positive reflector mode. As a results, 200 shots were accumulated for each spectrum obtained from the Voyager-DE STR and 1000 shots from the TOF/TOF. All MS/MS data were acquired using the default 1 kV MS/MS method installed according to the instruction. Proteins from the MS/MS were confirmed using the Protein Prospector (http://prospector.uscf.edu), the Proteomic Solution 1 system (Applied Biosystems), and the JGI database (http://genome.jgi.doe.gov/). In other words, a total of 150 peaks were systematically submitted for database searching against a small database which contains the sequences of protein internal standards alone.

**Supplementary References**

1. Bak JS, Kim MD, Choi I-G, Kim KH. 2010. Biological pretreatment of rice straw by fermenting with *Dichomitus squalens*. N Biotechnol 27:424–434.
2. Perie F, Gold M. 1991. Manganese regulation of manganese peroxidase expression and lignin degradation by the white rot fungus *Dichomitus squalens*. Appl Environ Microbiol 57:2240–2245.
3. Teunissen PJM, Field JA. 1998. 2-Chloro-1,4-dimethoxybenzene as a novel catalytic cofactor for oxidation of anisyl alcohol by lignin peroxidase. Appl Environ Microbiol 64:830–835.
4. Kersten PJ, Kirk TK. 1987. Involvement of a new enzyme, glyoxal oxidase, in extracellular H_2_O_2_ production by *Phanerochaete chrysosporium*. J Bacteriol 169:2195–2201.
5. Orth AB, Denny M, Tien M. 1991. Overproduction of lignin-degrading enzymes by an isolate of *Phanerochaete chrysosporium*. Appl Environ Microbiol 57:2591–2596.
6. Bourbonnais R, Paice MG. 1988. Veratryl alcohol oxidases from the lignin-degrading basidiomycete *Pleurotus sajor-caju*. Biochem J 255:445–450.
7. Muheim A, Waldner R, Leisola MSA, Fiechter A. 1990. An extracellular aryl-alcohol oxidase from the white-rot fungus *Bjerkendera adusta*. Enzyme Microb Technol 12:204–209.
8. Bao W, Lymar E, Renganathan V. 1994. Optimization of cellobiose dehydrogenase and β-glucosidase production by cellulose-degrading cultures of *Phanerochaete chrysosporium*. Appl Microbiol Biotechnol 42:642–646.
9. Fiehn O, Kopka J, Trethewey RN, Willmitzer L. 2000. Identification of uncommon plant metabolites based on calculation of elemental compositions using gas chromatography and quadrupole mass spectrometry. Anal Chem 72:3573–3580.
10. Bradford MM. 1976. A rapid and sensitive method for the quantitation of microgram quantities of protein utilizing the principle of protein-dye binding. Anal Biochem 72:248–254.
11. Shevchenko A, Wilm M, Vorm O, Mann M. 1996. Mass spectrometric sequencing of proteins silver-stained polyacrylamide gels. Anal Chem 68:850–858.

**SUPPLEMENTARY TABLE LEGENDS**

**Table S1.** Information of the adopted cellular proteins correlated with lignocellulolytic regulatory network during optimized *D. squalens* biodegradation.

**Table S1.** Information of the adopted cellular proteins correlated with lignocellulolytic regulatory network during optimized *D. squalens* biodegradation.

| **Section**^a^ | **JGI ID**^a^ | **Location**^a^ | **Putative function**^a^ | ***pI*/MW** | **Sequence**^a^ | **Cov.^b^** |
| --- | --- | --- | --- | --- | --- | --- |
| FC1 | 23624 | scaffold_74:47093-58635 (+) | Manganase peroxidase | 4.56/82.56 | RLLSDQVIAR | 70 |
| FC3 | 30327 | scaffold_8:700531-702559 (+) | Src homology-3 | 5.46/13.92 | RYEEDTVEGEPYYAQSNGR | 47 |
| FC1 and FC2 | 127252 | scaffold_15:447180-448777 (-) | Polysaccharide deacetylase | 5.48/100.38 | KSAQATGSGDASHNGAGAR | 37 |
| Unclassified | 137219 | scaffold_18:125989-127734 (+) | Hypothetical protein | 7.73/42.45 | KHTDWNGAFGVLESCADVCR | 45 |
| FC3 and FC4 | 159581 | scaffold_3:1656080-1662069 (-) | Ras GTPase | 4.88/41.32 | KPGGTVVEGTAGNTGIGLAHVCR | 30 |
| FC1 and FC2 | 165178 | scaffold_1:2892988-2895400 (-) | Glycoside hydrolase, family 28 | 5.42/26.51 | KCWDGLFVPTLK | 41 |
| FC1 and FC2 | 179533 | scaffold_8:180320-187544 (-) | Alpha/beta hydrolase fold-3 | 5.16/59.23 | KYMACALLYR | 80 |

^a^Information of *D. squalens* proteome were assigned based on the US Department’s Joint Genome Institute database.

^b^Sequence coverage (%) in peptide mass fingerprinting.
